# Sympathetic Ophthalmia*Synopsis of paper by Dr. J. M. Woodson, read at Waco meeting, Central Texas Medical Association, July 11, 1899. Reported by Dr. N. A. Olive, for Texas Medical Journal.

**Published:** 1899-09

**Authors:** 


For the Texas Medical Journal.

### Sympathetic Ophthalmia.*

This is an inflammation, usually plastic, may be serous, and affects the iris, ciliary body and choroid of one eye. Has its origin in traumatic inflammation of same parts of other eye.

Two factors constitute the disease: First, traumatic inflammation of one eye; second, plastic inflammation of the inner coat of the other eye. And to these may be added, time, for a limited period always elapses before the development of sympathetic disease. These are the three fundamental elements of true sympathetic ophthalmia. The absence of one would render diagnosis doubtful. Presence of all three would hardly warrant any other interpretation.

CAUSE. Penetrating or lacerated wounds play the most important part in causation. A blow on the eye without rupture of the tunics, or wounds from chemicals may excite sympathetic inflammation in the other eye. Intra-ocular tumors, such as glioma and sarcoma may cause it. Any condition producing irido-cyclitis (an inflammation of iris and ciliary body) may cause it. Injuries near the margin of the cornea are the most serious.

Traumatic cataract when caused from wound in the ciliary region adds to the gravity of wound by inflamed lens pressing against the inflamed and injured ciliary body and acting as a foreign body in the eye. Traumatic cataract will not produce sympathetic ophthalmia when iris and ciliary body are uninjured. The onset is generally insidious; blindness may come on with very little pain.

*Synopsis of paper by Dr. J. M. Woodson, read at Waco meeting, Central Texas Medical Association, July 11, 1899. Reported by Dr. N. A. Olive, for TEXAS MEDICAL JOURNAL.

One of the most frequent symptoms is the readiness with which the eye tires, when attempting to fix the vision on an object, no matter whether the object is large or small, far or near. If effort at fixation is continued, the eye will run water, the image become blurred and injection around the cornea become intense without symptoms to account for it.

The media are usually hazy. A view of the interior of the eye by means of the ophthalmoscope is impracticable.

Eye tension is variable; generally increased at beginning, and later the eye softens. The disease may run its course in a short time, causing total permanent blindness; or it may run a chronic course; after repeated exacerbations become arrested, leaving sufficient vision to enable patient to get around.

If a chronic course results, the pupil usually becomes occluded by an organized exudate which cuts off communication between the two chambers of the eye. In addition to this, the posterior surface of the iris becomes glued to the anterior surface of the capsule of the lens, thus disturbing the nutrition of the eye and causing atrophy.

Three weeks after the primary injury is received is the shortest time at which we may expect sympathetic symptoms. The longest period is uncertain. Most cases come on from three weeks to four months after injury. There are well authenticated cases coming on forty-five years after primary injury..

In Alt's table of statistics, $22\frac{3}{4}$ per cent. of cases came on between one and ten years after injury, 12 per cent. between ten and twenty years, and 13 per cent. between twenty and sixty years, and 53 per cent. before expiration of the first year. I do not think an atrophic eye free from pain, irritation and inflammation, can give rise to inflammation in its fellow.

If an atrophic eye is insensible to moderate pressure, there is no danger of sympathetic disease.

THE TREATMENT SHOULD BE PROPHYLACTIC: Remove the injured eye before symptoms of irritation occur in its fellow, and you need not fear sympathetic trouble.

An eye capable of causing sympathetic ophthalmia is dangerous, and should be removed, whether the eye is entirely sightless or not.

### DISCUSSION.

DR. REILY: I have never seen a case of sympathetic ophthalmia where it was necessary to remove the eye. All writers put a very grave phase on the trouble. I have seen cases in the St. Louis clinics where the eye was lost.

I think, as to infection of other eye, the infective principle or germ may as well travel down the optic nerve and up to the other eye. But with gonorrheal ophthalmia we know the infection is from the outside.

DR. SEARS: I have not much confidence in sympathetic ophthalmia. I think when it takes from ten to forty years to get from one eye to the other, the well eye is slow in expressing its sympathy for its fellow.

DR. WOODSON: There are three ways in which sympathetic ophthalmia may be caused. First: By way of optic nerve, through nerve action; not by there never being a road for the germs to travel over. I never heard of such a theory. Second. By ciliary nerves. Third. Through the blood vessels. Sympathetic ophthalmia is not generally slow to go from one eye to the other. We should never lose sight of its possibility. A sightless eye should be removed if there is any indication of sympathetic ophthalmia.

DR. REILY: I did not intend to say that the germs would or could travel over the optic nerves from one eye to the other. The gentlemen must have misunderstood me. The secretary will please make the correction.

